# Characterization of Newly Isolated Lytic Bacteriophages Active against *Acinetobacter baumannii*

**DOI:** 10.1371/journal.pone.0104853

**Published:** 2014-08-11

**Authors:** Maia Merabishvili, Dieter Vandenheuvel, Andrew M. Kropinski, Jan Mast, Daniel De Vos, Gilbert Verbeken, Jean-Paul Noben, Rob Lavigne, Mario Vaneechoutte, Jean-Paul Pirnay

**Affiliations:** 1 Laboratory for Molecular and Cellular Technology (LabMCT), Queen Astrid Military Hospital, Brussels, Belgium; 2 Eliava Institute of Bacteriophage, Microbiology and Virology, Tbilisi, Georgia; 3 Laboratory for Bacteriology Research (LBR), Faculty Medicine & Health Sciences, Ghent University, Ghent, Belgium; 4 Laboratory of Gene Technology, Faculty of Bioscience Engineering, Katholieke Universiteit Leuven, Leuven, Belgium; 5 Laboratory for Foodborne Zoonoses, Public Health Agency of Canada, Guelph, Ontario, Canada; 6 Department of Molecular and Cellular Biology, University of Guelph, Guelph, Ontario, Canada; 7 Electron Microscopy Unit, Veterinary and Agrochemical Research Centre, Brussels, Belgium; 8 Department of Pathology, Bacteriology and Poultry Diseases, Ghent University, Merelbeke, Belgium; Naval Research Laboratory, United States of America

## Abstract

Based on genotyping and host range, two newly isolated lytic bacteriophages, myovirus vB_AbaM_Acibel004 and podovirus vB_AbaP_Acibel007, active against *Acinetobacter baumannii* clinical strains, were selected from a new phage library for further characterization. The complete genomes of the two phages were analyzed. Both phages are characterized by broad host range and essential features of potential therapeutic phages, such as short latent period (27 and 21 min, respectively), high burst size (125 and 145, respectively), stability of activity in liquid culture and low frequency of occurrence of phage-resistant mutant bacterial cells. Genomic analysis showed that while Acibel004 represents a novel bacteriophage with resemblance to some unclassified *Pseudomonas aeruginosa* phages, Acibel007 belongs to the well-characterized genus of the *Phikmvlikevirus*. The newly isolated phages can serve as potential candidates for phage cocktails to control *A. baumannii* infections.

## Introduction

Multidrug-resistant (MDR) *Acinetobacter baumannii* is a relatively newly emerged pathogen, notorious for its role in nosocomial wound infections [1]–[3]. The ability to form biofilms and extended survival on environmental surfaces along with broad resistance to antibiotics puts *A. baumannii* on the list of the medically most important pathogens [4]. The known risk factors for *A. baumannii* colonization or infection include prolonged hospitalization, intensive care unit (ICU) admission, recent surgical procedures, parenteral nutrition, invasive procedures, nursing home residence, and previous broad-spectrum antibiotic use [5]–[9].

In comparison to other clinically relevant bacteria, *Acinetobacter* spp. can develop antibiotic resistance extremely rapidly due to long-term evolutionary exposure to soil organisms that produce antibiotics [10]. Many *A. baumannii* strains are characterized by an impressive number of acquired mechanisms of resistance to antibiotics, including enzymatic inactivation, modification of target sites, active efflux and decreased influx of drugs [11]. In addition, *A. baumannii* has a naturally occurring “*bla*_OXA-51_-like” carbapenem-hydrolyzing class D β-lactamase (CHDL) gene, intrinsic to this species, although phenotypic resistance is not associated only with the presence of this gene and normally depends on other genetic determinants as well [12].

Treatment of MDR strains of *A. baumannii* is a challenging issue for modern medicine. Carbapenems are the antibiotics of the first choice against multidrug resistant *A. baumannii* infections. However, carbapenem-resistant *A. baumanniii* are being increasingly reported worldwide [6], [13]. Other therapeutic options include sulbactam, aminoglycosides, polymyxins and tigecycline. Recently, high resistance rates to tigecycline and polymyxins were also reported [14]–[16].

Bacteriophages active against MDR strains of *A. baumannii* could be considered as a potential solution to meet the challenges posed by this pathogen. Indeed, a number of new phages carrying potential to combat infections caused by *A. baumannii* strains have been recently reported [17]–[24].

In our study, we isolated and characterized two highly lytic bacteriophages: myovirus vB_AbaM_Acibel004 and podovirus vB_AbaP_Acibel007 (from here on shortened to Acibel004 and Acibel007, respectively), named in accordance with the proposed nomenclature for bacteriophages by Kropinski *et al*. [25]. The phages were characterized, according to criteria considered as essential for potential therapeutic phages and both phages proved to meet all of them.

## Materials and Methods

### Bacterial strains

In total, 34 strains of *Acinetobacter* spp. were used in the study (Table S1). Twenty one *A. baumannii*, three *A. pittii* and one *A. nosocomialis* strains were isolated from successive outbreaks in the Burn Wound Center of the Queen Astrid Military Hospital (Brussels, Belgium). Eight strains were received from Leiden University Medical Center, the Netherlands: one *A. pittii* strain, one *A. nosocomialis* strain and six *A. baumannii* strains including representatives of European epidemic clones I, II and III. The *A. baumannii* T strain NCTC 13423 was obtained from Public Health England, UK [12]. Initial identification and antibiotic susceptibility profile detection of the strains were performed by the VITEK 2 system (BioMérieux, France) using ASTN155 card for Gram-negative bacteria. For the precise identification, partial sequencing of the *rpoB* gene was performed [26]. All strains were typed by rep-PCR using the DiversiLab *Acinetobacter* 3.4 kit (BioMérieux, France). A cluster of closely related isolates was defined as isolates sharing ≥95% similarity [27]. All strains were screened for the presence of *bla*_OXA-51_-like and *bla*_OXA-23_-like carbapenemases encoding genes in their genomes by Multiplex-PCR (M-PCR) [28].

### Phage isolation, purification and propagation

Ghent University Hospital (GUH) waste water samples were used for isolation of active phages by culture-enrichment method described in Merabishvili *et al*. [29], with slight modifications. Briefly, 15 ml of 10× concentrated LB broth (Becton Dickinson, Erembodegem, Belgium) was mixed with 135 ml sample water and 1 ml (10^8^ cfu/ml) of one/different combinations of test strains in a 200 ml bottle. The mixture was incubated at 32°C overnight. Subsequently, 3 ml of chloroform was added and the bottle was further incubated at 4°C for 2 h. The parallel streaks and agar-overlay methods [29] were used to check the lysates for the presence of phages and subsequent isolation of pure phage particles. Plaques with different morphology were touched with a sterile pipette tip, inoculated into 2 ml of sterile LB broth and incubated at 37°C for 2 h. Each phage separation procedure was repeated 10 times to ensure single phage suspensions.

High titer bacteriophage stocks were prepared by the agar overlay method [29]. The *A. baumannii* strain 070517/0072 served as a host strain for the propagation of the Acibel004 and Acibel007 phages. Further phage particles in the obtained lysates were precipitated using PEG 8000 (8%, wt/vol) + 1 M NaCl according to Sambrook and Russel [30]. Phages for proteomic analysis were purified by centrifugation in CsCl gradients at 140,000×*g* for 3 h [30].

### Host range analysis and determination of Efficiency of Plating (EOP)

The parallel streaks method [29] was used to detect the susceptibility of the 34 bacterial strains for the two phages. All susceptible strains revealed by the parallel streaks method were titered against the phages to detect the EOP and the ability of the phage to propagate in the strain. EOP was calculated as the ratio of titer on the test strain to the titer on the host strain.

### Transmission electron microscopy

Bacteriophage particles were analyzed by transmission electron microscopy as described by Imberechts *et al*. [31]. Briefly, suspensions were brought on carbon and pioloform-coated grids (Agar Scientific, Stansted, UK), washed with water and negatively stained with 2% uranyl acetate (Agar Scientific) in water and analyzed using a Technai Spirit transmission electron microscope (FEI, Eindhoven, The Netherlands), operating at 120 kV. Micrographs were recorded using a bottom-mounted digital camera (Eagle, 4X4K, FEI).

### Phage adsorption and one-step growth parameters

Exponentially grown *A. baumannii* 070517/0072 cells were mixed with each phage at a MOI of 0.001 and incubated at 37°C. Aliquots of 50 µl were removed after 3, 5, 8, 10, 15 and 20 min and diluted 100 times in 4.45 ml LB broth and 0.5 ml chloroform. The tubes were incubated for 30 min at room temperature and subsequently titrated to detect the number of non-adsorbed phages. The rate of adsorption was determined according to Adams [32]. The adsorption curves were constructed based on the ratio of non-adsorbed phage at different time intervals over the initial phage number.

In the one-step growth experiment, *A. baumannii* 070517/0072 (10^8^ cfu/ml) was infected with each phage at a MOI of 0.001. The adsorption process was allowed to occur during 8 min at 37°C. The mixture was centrifuged (13,000×*g*, 1 min), and the pellet resuspended in 10 ml of fresh LB broth and incubated for 1.5 h at 37°C. The samples were taken at intervals of 3 minutes and titrated. The latent period was defined as the interval between adsorption of the phages to the bacterial cells and the release of phage progeny. The burst size of the phage was determined as the ratio of the final number of free phage particles to the number of infected bacterial cells during the latent period.

### Frequency of occurrence of phage-resistant mutant bacterial cells

The frequency of occurrence of phage-resistant mutant bacterial cells was detected by the method described in Adams [32]. Briefly, 1 ml of bacterial culture at the concentration of 10^8^ cfu/ml was mixed with the appropriate volume of phage lysate at a MOI of 100. After 10 min of incubation at 37°C, 100 µl of the mixture was spread on LB plates and phage-resistant colonies were enumerated after overnight incubation at 32°C. The emerged colonies were isolated twice to assure phage-free bacterial cultures. Further the isolated bacterial cultures were tested against phages by parallel streaks method to confirm their true resistance. The experiments were performed in triplicate and mean values calculated.

### Stability of phage activity in broth culture

Stability of the activity of each phage separately and in mixture with the other phage in LB broth was studied according to the method of Appelmans [32]. Briefly, serial tenfold dilutions of each phage and of the mixture of phages, with initial concentration of 10^9^ pfu/ml, were prepared in 5 ml LB broth starting from 10^−2^ dilution. *A. baumannii* 070517/0072 bacterial culture was added to each dilution with a final concentration of 10^5^ cfu/ml, thereby multiplicity of infection (MOI) ranged from 100 to 0.00001. Bacterial growth and phage activity was monitored by measuring OD_600_ after 24 and 48 h of incubation at 37°C.

### Fluorescent RFLP analysis of phage genome

fRFLP analysis on phage genomes was performed according to Merabishvili *et al*. [34] with slight modifications: fluorescent primer HhaI+O was labeled with FAM (5-carboxyfluorescein) and PCR products were analyzed in ABI 3130xl Genetic Analyzer. Comparison of digitized fingerprints was carried out by the in-house program Basehopper, available at www.basehopper.be. A similarity tree was constructed by Neighbor joining using the programs PHYLIP [http://evolution.genetics.washington.edu/phylip.html] and TreeView [http://taxonomy. zoology.gla.ac.uk/rod/treeview.html].

### DNA sequencing and genome annotation

DNA from high titer phage stocks was isolated by the classic phenol-chloroform extraction method, according to Sambrook & Russell [30]. The procedure was preceded by DNase I and RNase A treatment at the concentrations of 10 µg/ml and 55 µg/ml, respectively, to remove bacterial nucleic acids. The sequences were determined by pyrosequencing (454 technology). ORF Finder [www.ncbi.nlm.nih.gov/projects/gorf, accessed on July 20, 2013], GeneMark [35] and Glimmer [36], [37] were used to predict open reading frames (ORFs) in the phage genomes. Putative functions were assigned to the corresponding gene products based on the results of a protein BLAST (version 2.2.26) [38], [39] and HHpred [40]. The search for signal peptide cleavage sites and transmembrane helices was performed using SignalP (4.1) [41] and TMHMM (2.0) [42], respectively. The presence of tRNA-encoding genes was determined by Aragorn (1.2.36) [43] and tRNAscan-SE (1.21) [44]. Conserved regulatory elements, such as putative host- and phage-specifc promoters and Rho-factor independent terminators were searched by MEME/MAST (4.9.0) [45], ARNOLD [46], [47] and PHIRE [48]. All these steps were followed by manual verification. Promoters were evaluated on the presence of consensus sequences at −35 and −10 upstream, terminators were scored based on the presence of a strong palindromic sequence, followed by a U-rich stretch and an estimated free energy value for stem-loop formation of −8 at max. Based on the annotated phage genomes, GenBank files were created with Sequin [www.ncbi.nlm.nih.gov/projects/Sequin/] and subsequently used to construct the genomic maps with the help of EasyFig v. 2.1 [49].

### Comparison of phage genomes and phylogenetic study

DNA homology of phage genomes on nucleotide level was compared by EMBOSS stretcher [50], [51]. A phylogenetic analysis based on RNA-polymerase was performed by the maximum-likelihood method, using MEGA5 [52]. The sequences of 19 phage RNA-polymerases with the following accession numbers were used: *A. baumannii* phages AB3 (NCBI accession No. YP_008060148) and phiAB1 (ADQ12732), *Vibrio parahaemolyticus* phage VP93 (YP_002875649), Enterobacteria phages T3 (CAC86264), T7 (AAA32569) and SP6 (NP_853568), *Yersinia* phage φA1122 (AAP20500), *Erwinia amylovora* ERA103 (YP_001039639), *Prochlorococcus* phage P-SSP7 (YP_214191), *Ralstonia* phage RSB1 (YP_002213715) *Pseudomonas putida* phage gh-1 (NP_813747), *Pseudomonas aeruginosa* phages LKA1 (CAK25005), phi-2 (YP_003345489), LKD16 (YP_001522818), phikF77 (YP_002727849), phiKMV (NP_877465), PT5 (ABW23108), PT2 (ABY70996) and LUZ19 (YP_001671971).

### Proteome analysis of phages

Proteome analysis was performed on phage proteins obtained by methanol-chloroform extraction (1∶1:0.75, v/v/v) from cesium chloride purified phage suspension, according to Moak & Molineux [53]. Protein pellets were resuspended in SDS-PAGE buffer (1% sodium dodecyl sulphate (SDS), 6% sucrose, 100 mM 1,4-dithiothreitol (DTT), 10 mM Tris, 0.0625% bromophenol blue, pH 6.8) and boiled at 95°C for 3 minutes. The samples were loaded on standard 12% polyacrylamide gel. After visualization by staining with Simply Blue TM Safe Stain (Invitrogen Ltd, Paisley, UK), the different protein bands were isolated. An in-gel digestion of the proteins was performed with trypsin by overnight incubation at 37°C. The resulting peptides were extracted from the gel by three successive rounds of sonication and analyzed using electrospray ionization-tandem mass spectrometry (MS/MS) as described previously by Lavigne *et al*. [54]. The obtained spectra were screened against a database containing all predicted protein sequences. The used identification parameters were ‘minimum of two peptides found per protein’, with a combined ‘protein identification probability’ of at least 99.8% and a ‘best peptide identification probability’ of 95%.

### Nucleotide sequence accession numbers

The genome sequences of Acibel004 and Acibel007 phages were submitted to GeneBank. Assigned accession numbers are KJ473422 for Acibel004 and KJ473423 for Acibel007.

### Ethics statement

No official permission was required to make waste water sampling in Ghent University Hospital.

## Results and Discussion

*A. baumannii* has been listed by the Infectious Diseases Society of America (IDSA) as one of the six top-priority dangerous microorganisms [55]. It has even been suggested that we are closer to the end of the antibiotic era with *Acinetobacter* than with methicillin-resistant *Staphylococcus aureus* [56]. The increased occurrence of *A. baumannii* MDR strains in the Burn Wound Center of Queen Astrid Military Hospital (Brussels, Belgium) and in health care settings worldwide in general [9], [16] prompted us to search for alternative solutions to the problem, such as bacteriophages.

### Establishing the *A. baumannii* phage collection and characterization of bacterial strains

A total of 26 phages were isolated from the GUH wastewater samples using various combinations of *A. baumannii* clinical strains (in total 28 strains). Based on genotyping and host range (see below), Acibel004 and Acibel007 phages were selected for further characterization. *A. baumannii* clinical strain 070517/0072, isolated from the nose of a patient in Queen Astrid Military Hospital, showed the highest susceptibility to both phages and was chosen as the host strain for further propagation of the phages.

The host range of the newly isolated phages was studied on a collection of strains including the 28 clinical *A. baumannii* strains and other representatives of the genus *Acinetobacter*, in particular four *A. pittii* and two *A. nosocomialis* strains of clinical origin (Table S1). All bacterial strains used in the study were characterized according their susceptibility profiles towards four classes of antibiotics, i.e. aminoglycosides, quinolones, carbapenems and polymyxins (with colistin as the only representative). Generally, carbapenems and polymyxins are considered as last resort antibiotics against MDR *A. baumannii* infections. At the same time well-known nephrotoxicity and neurotoxicity of polymyxins makes their application less favorable and is regarded as a last remaining option. The most common mechanism of *A. baumannii* resistance to carbapenems involves the ability to synthesize carbapenem-hydrolyzing class D β-lactamases (CHDL). The CHDLs of *A. baumannii* are divided into five phylogenetic subgroups: OXA-23-like, OXA-40-like, OXA-51-like, OXA-58-like and OXA-143-like carbapenemases. Recently, a new OXA-235-like CHDL has been reported [57]. The presence of a *bla*_OXA-51_-like CHDL gene is considered intrinsic for *A. baumannii,* because of its chromosomal location and its presence in the majority of isolates of *A. baumannii*. The genes of the other four established CHDL groups are mostly found on plasmids and are considered as acquired genes. Indeed, the strains of *A. pittii* and *A. nosocomialis* used in the study, phenotypically sensitive to all representatives of four tested classes of antibiotics, were negative for *bla*_OXA-51_-like CHDL encoding genes, while all 28 strains of *A. baumannii* comprised *bla*_OXA-51_-like CHDL encoding gene in their genomes (Table S1). However, the presence of the *bla*_OXA-51_-like CHDL gene is not an unambiguous indicator of carbapenem-resistance in *A. baumannii* strains, because the resistance occurs only when the insertion sequence IS*Aba1* is present upstream of the gene [28]. According to Yang *et al*. [5], acquired subgroup of *bla*_OXA-23_-like carbapenemase genes are more likely to contribute to the carbapenem resistant phenotype of strains, although silent presence of the gene due to the lack of insertion sequences is not unusual among *A. baumannii* strains of clinical origin [58], [59]. Two thirds of *A. baumannii* strains in the study (18 out of 28) were resistant to carbapenems, and 16 out of these 18 carbapenem resistant strains were positive for the *bla*_OXA-23_-like carbapenemase gene presence. Exceptions included: strain 080502/0184 positive for the *bla*_OXA-23_-like CHDL gene but sensitive to carbapenem and two strains (NCTC 13423 and LUH 5875 (European epidemic clone III)) negative for the *bla*_OXA-23_-like CHDL gene but resistant to carbapenem. Carbapenem-susceptibility of the strain 080502/0184 can be explained by the absence of insertion sequence IS*Aba1* in upstream position of *bla*_OXA-23_-like CHDL gene as mentioned above [58], [59]. In case of the strains NCTC 13423 and LUH 5875 (European epidemic clone III) it is known that carbapenem resistance is expressed due to the insertion sequence IS*Aba1* linked to *bla*_OXA-51_-like CHDL gene in upstream position [28], [60]. Most of the studied *A. baumannii* strains were also characterized by resistance to aminoglycosides and quinolones and all of them revealed susceptibility to colistin.

All 34 strains of *Acinetobacter* spp. were typed by rep-PCR (DiversiLab Acinetobacter 3.4 kit BioMérieux) and 15 different profiles were identified (Table S1). Four profiles within the *A. baumannii* were represented with more than one strain. Within each profile, the strains shared several features, including the presence or absence of the *bla*_OXA-23_-like and *bla*_OXA-51_-like CHDL genes, and antibiotic and phage susceptibility. However, limited variability was observed in three groups representing different rep-profiles. Only one strain out of three with rep-profile 4 revealed resistance to carbapenem and susceptibility to phage Acibel004. Two strains with rep-profile 8 differed from each other by susceptibility to quinolones and phage Acibel004 (Table S1). The rep-profile 11 was the largest group and comprised 12 strains. One strain from this group lacked the *bla*_OXA-23_-like CHDL gene. Two strains, including the strain without *bla*_OXA-23_-like CHDL gene, showed carbapenem-susceptibility, while the second one was resistant to Acibel004. Acibel007 was only able to adsorb but not to propagate on two other strains from the group and the rest 9 strains were susceptible to both phages.

### Host range study of phages

All six non *A. baumannii* strains (4 *A. pittii* and 2 *A. nosocomialis*) were resistant to the newly isolated phages Acibel004 and Acibel007. However, Acibel004 still showed weak adsorption on all strains except for one *A. pittii* strain.

**Figure 1:**
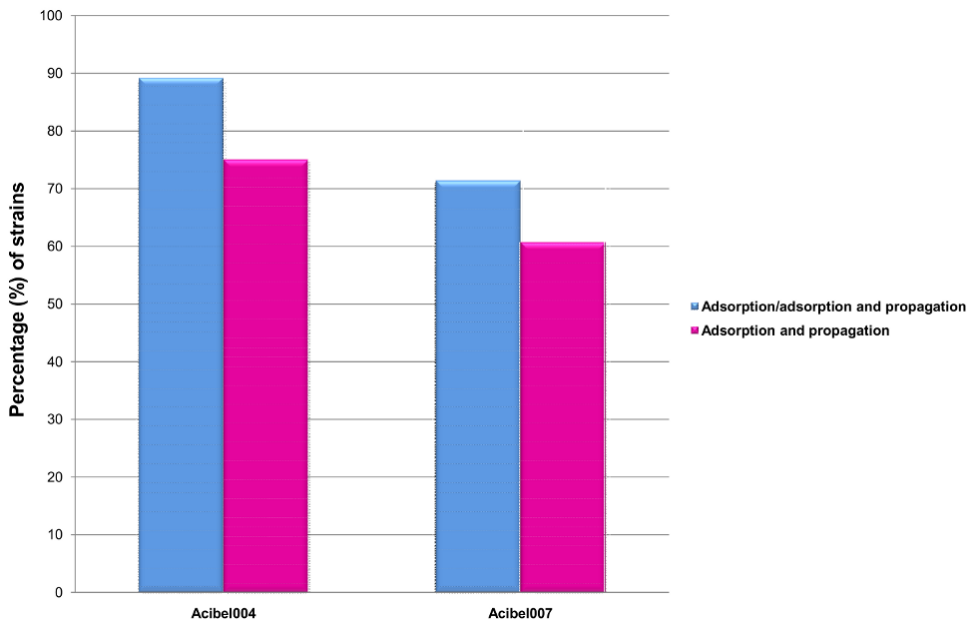
Activity of phages Acibel004 and Acibel007 on 28 *A.* *baumannii* clinical strains.

Both phages were able to propagate on approximately half of the tested *A. baumannii* strains (15 out of 28), each phage was able either to adsorb and/or propagate on 11 strains but 2 strains showed complete resistance to both phages. Phage Acibel004 is able to adsorb on 89.2% of 28 tested *A. baumannii* strains and to propagate on 75.0% of them, whereas phage Acibel007 adsorbs to 71.4% and propagates on 60.7% of the strains (Fig. 1). Based on the host range studies, the newly isolated phage Acibel007 can be considered as strictly specific towards *A. baumannii*, while Acibel004 is a phage which propagates only in *A. baumannii* strains but still maintains weak adsorption activity on the strains of *A. pitti* and *A. nosocomialis*. At the same time, no correlation was detected between phage susceptibility, antibiotic resistance and rep-pcr profile affiliation of the clinical strains used in the study.

The range of EOP for Acibel004 phage on susceptible *A. baumannii* strains varied between 1.0–1.0^−5^, and for Acibel007 between 6.25^−1^–6.25^−2^ (Table S1). Therefore, taking into account the measured EOP, Acibel007 can be considered as a phage with higher lytic activity than Acibel004, despite its more limited host range.

### Phenotypic characterization of phages

**Figure 2:**
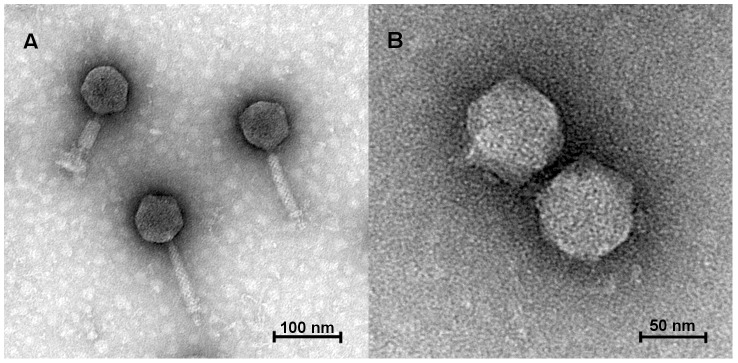
Transmission electron micrographs of phages. A) Acibel004, member of the *Myoviridae* family; B) Acibel007, member of the *Podoviridae* family.

Acibel004 and Acibel007 phages form clear plaques on the host strain, differing in size. Acibel004 phage forms plaques with a diameter of 1–2 mm, compared to 3–5 mm for Acibel007. Phage particle morphology examined by TEM revealed that Acibel004 and Acibel007 can be assigned to different families of the order *Caudovirales* (Fig. 2). Acibel004, with a 70 nm icosahedral head and a 105 nm long contractile tail, is classified as morphotype A1 of the *Myoviridae*, while Acibel007, with a 60 nm head and a 10 nm long noncontractile tail, belongs to morphotype C1 of the *Podoviridae*.

The frequency of appearance of phage-resistant mutants in phage-sensitive strains is one of the characteristics of major importance for therapeutic bacteriophages. In case of the newly isolated phages Acibel004 and Acibel007, the mutation frequency parameters in the host strain *A. baumannii* 070517/0072 are 1.2±0.5×10^−7^ and 3.0±1.5×10^−6^, respectively. These figures indicate a low ability of the strain to obtain phage-resistance mutants.

**Figure 3:**
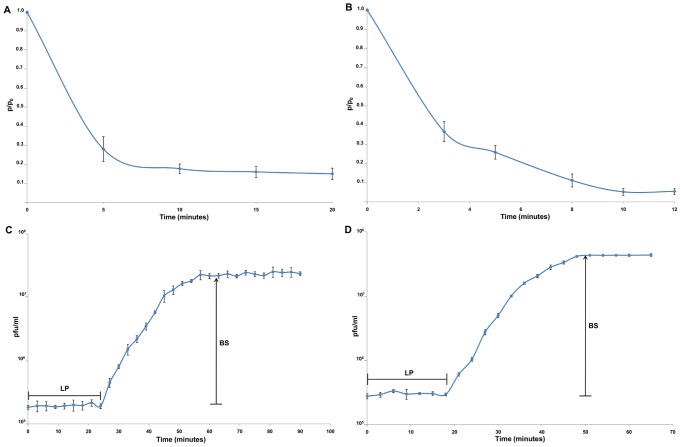
Infection parameters of phages. A) Adsorption curve of Acibel004; B) Adsorption curve of Acibel007; C) Single-step growth curve of Acibel004; D) Single-step growth curve of Acibel007. P-number of non-adsorbed phages, P_0_- initial number of phages, LP-latent period, BS-burst size. The results are the mean values of three independent tests. Standard deviations are indicated.

The phage adsorption and single-step growth processes were studied to obtain an overall impression of the phage infection cycle. Acibel004 phage achieves a maximal adsorption of 85% within 15 min (Fig. 3A). A maximum of 95% of the Acibel007 phage particles absorb on the host strain within 10 min (Fig. 3B). Adsorption velocity constants for Acibel004 and Acibel007 equal to 1.2×10^−9^ and 1.7×10^−9^ ml/min, respectively.

The single-step growth experiments were performed for determination of latent periods and burst sizes of phages, as important characteristics of phage infection process. The latent period of Acibel004 is 27 min and the average burst size is 125 phage particles per infected cell. Acibel007 is characterized with a shorter latent period of 21 min and a bigger burst size of 145 phage particles per infected host cell (Fig. 3C and 3D). The obtained physiological parameters of the newly isolated phages characterize them as phages with high activity against the susceptible strains.

### Activity of phages in broth culture

According to production guidelines of different phage preparations [61]–[64], which existed in Soviet Union in the past and exist in Georgia and Russia at present, phage titer of therapeutic preparations is required to be expressed as activity of phages in broth measured by the Appelmans’ method [33]. Stable activity of phages in broth cultures represents their ability to overcome the growth of phage-resistant mutants and gives an indication on effective titer of therapeutic preparations to be used in future.

**Figure 4:**
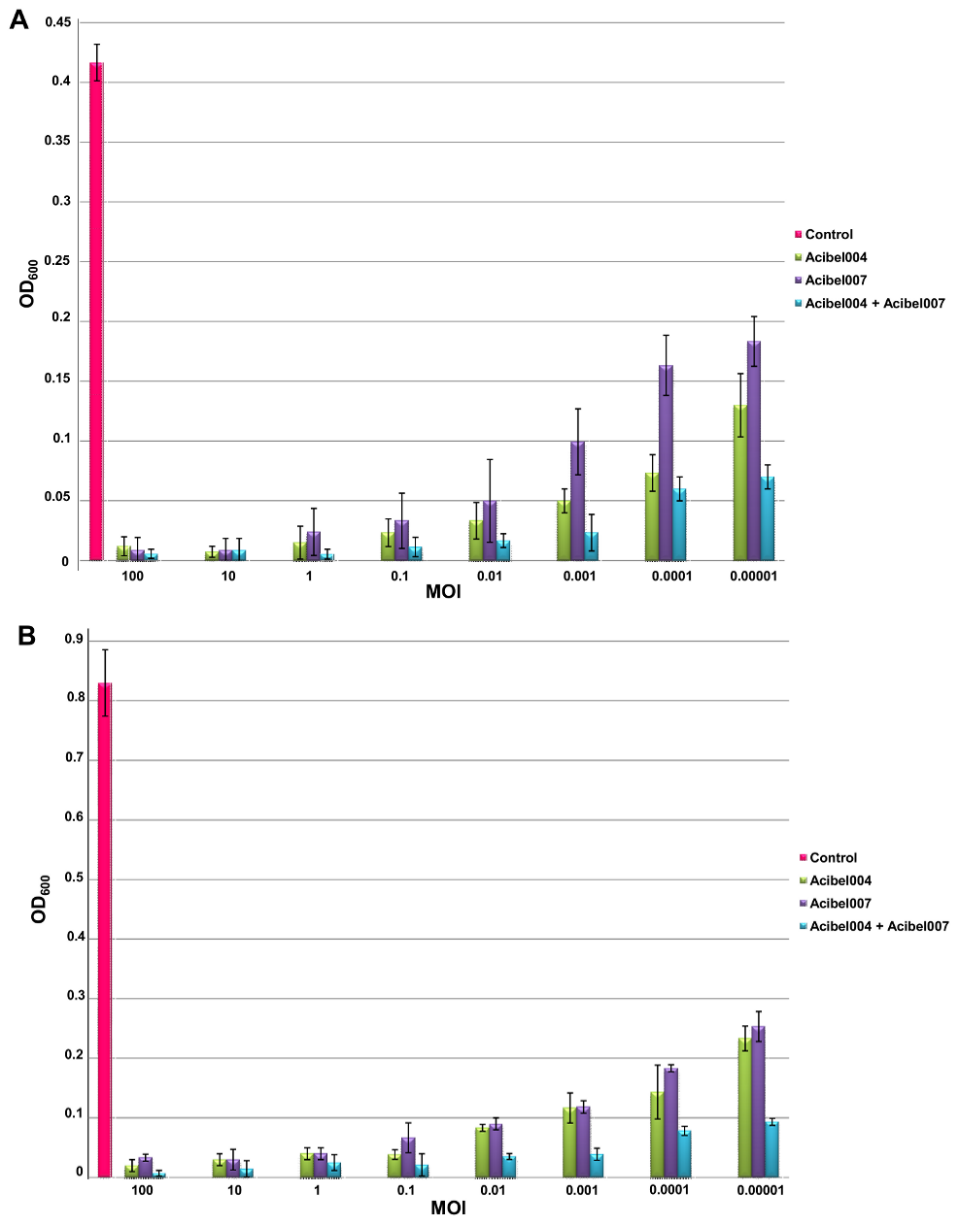
Stability of activity of phages in LB broth media. A) Study of phage activity after 24 hours; B) Study of phage activity after 48 hours. Control: non-infected bacterial culture of *A. baumannii* strain 070517/0072. The results are the mean values of three independent tests. Standard deviations are indicated.

The ability of Acibel004 and Acibel007 to influence each other’s activity while cultured together was tested in LB broth by the Appelmans method along with assessment of the stability of activity for a 24–48 hour period (Fig. 4). Even at the lowest MOI of 0.00001, both phages succeed to maintain a lower OD_600_ value of the bacterial suspension, compared to the control, i.e. a non-infected bacterial culture: after 24 h of incubation. The OD_600_ value for Acibel004 equaled to 0.13±0.03, for Acibel007 to 0.18±0.02 and for the mixture of phages to 0.07±0.01, while the OD_600_ for the control corresponded to 0.42±0.02 (Fig. 4A). The mixture of both phages showed a relatively high activity in comparison to each phage separately at all tested MOI, even after 48 h of incubation, i.e. at MOI of 0.00001 the OD_600_ of Acibel004 and Acibel007 separately, equaled to 0.23±0.02 and 0.25±0.03, respectively, while the OD_600_ of both phages was 0.09±0.01 against 0.83±0.06 of the control (Fig. 4B). This finding indicates that a mixture of Acibel004 and Acibel007 is more effective than each phage separately. This could be explained by phages having different adsorption receptors on the bacterial cell wall, so bacterial cells have to accumulate different mechanisms of resistance.

Stable activity of each phage in broth expressed by maintaining OD_600_<0.1 after 48 h incubation at a minimum MOI of 0.01 and increased activity during joint culturing; indicate that these two phages are good candidates to be used in a phage cocktail together.

### Phage genome analysis and annotation

The genomes of the two newly isolated phages were typed by fRFLP [34] along with other representatives from a collection of *A. baumannii* bacteriophages, isolated during the same period. In total, 26 phage genomes were screened based on neighbor joining analysis and a phylogenic tree was constructed (Fig. S1). Acibel004 phage grouped within a large group of 15 myoviruses with very similar RFLP profiles, whereas Acibel007 grouped together with the only other podovirus presented in this collection, i.e. Acibel020. The results of fRFLP contributed to the selection of Acibel004 and Acibel007 as appropriate candidates for phage therapy and supported further detailed investigation of their genome and proteome, considering their largest host spectrum among other members of the genetically related phage groups (Fig. S2).

Sequencing of the genomes of Acibel004 and Acibel007 revealed that both phages have linear double-stranded DNA with a length of 99,730 and 42,654 bp, respectively. G+C content of the Acibel004 phage genome is 37.3%, and 41.2% for the Acibel007 genome, i.e. values similar to the host’s average genome G+C content of 40%. A high percentage of the genomes of these phages, 90.4 and 93.6% of Acibel004 and Acibel007, respectively, are represented by coding sequences.

**Figure 5:**
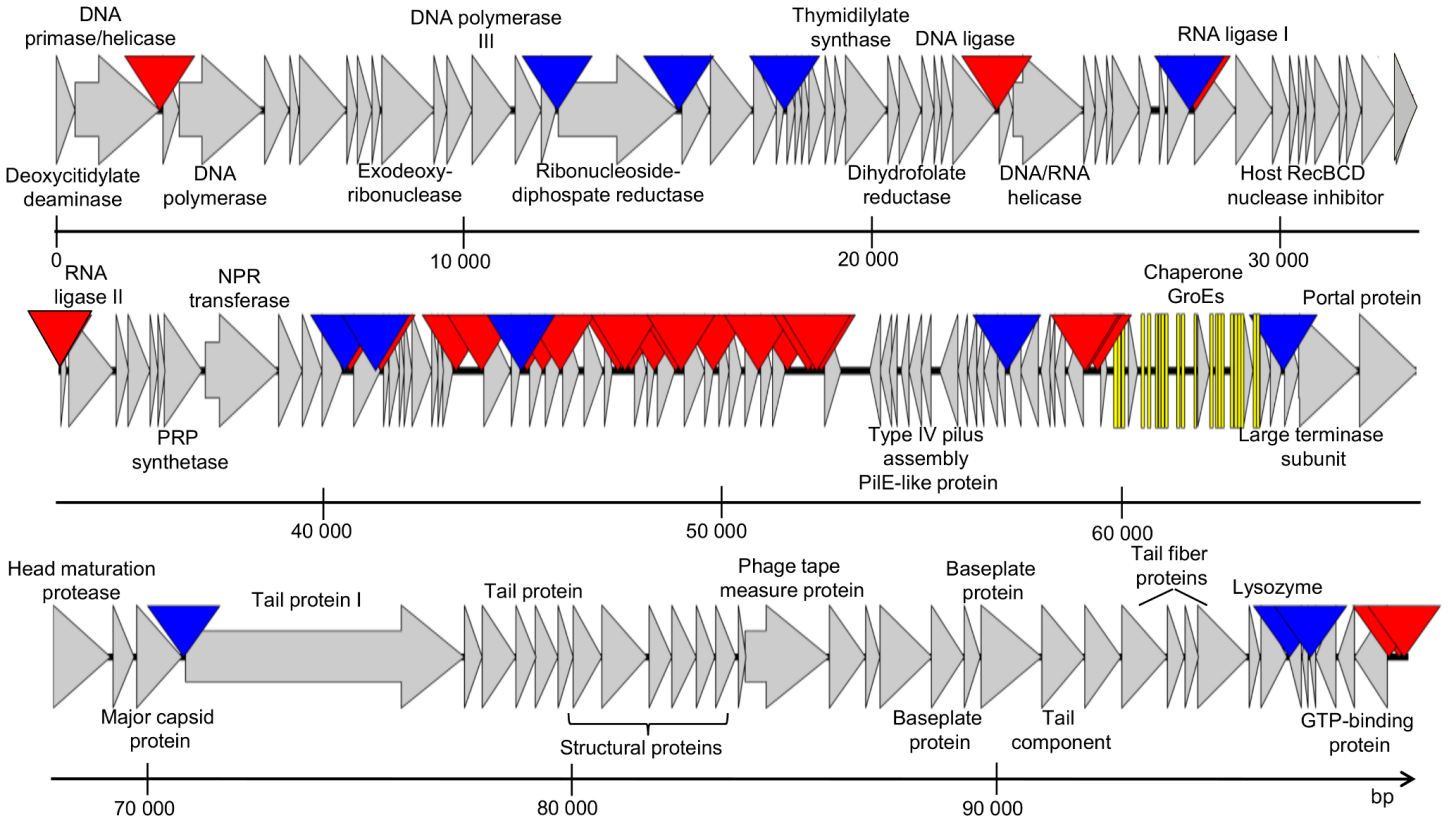
Genome map of Acibel004. tRNAs are indicated with yellow bars, putative host-specific promoters and Rho-factor independent terminators with red and blue triangles, respectively. PRP: phosphoribosylpyrophosphate, NPR: nicotinamide phosphoribosyl.

The genome of Acibel004 encodes 156 ORFs on both strands of the DNA molecule (Fig. 5 and Table S2), and 22 tRNAs, localized in the genomic region between 111 and 115 ORFs. Presence of tRNA genes in phage genomes, especially in virulent phages (e.g. T4 like phages) is quite common phenomenon [65], [66]. Bailly-Bachet *et al.* [66] observed that tRNAs that are kept in virulent phage genomes are those corresponding to codons abundant in the phage and rare in the host. We analyzed codon usage frequency in the genomes of Acibel004 and *A.baumannii* ATCC 17978 strain [http://www.kazusa.or.jp/codon/cgi-bin/showcodon.cgi?species=400667]. Most of the codons (16 out of 21) to which 22 phage-encoded tRNAs are matching, appeared to be presented with higher frequency in phage genome than in *A. baumannii* (Fig. S3). Genome ends of Acibel004 were identified to be not permutated (by Bal31 restriction, data not shown) but could not be determined by sequencing. Transmembrane domains were predicted in gene products of 21 ORFs (Table S2). The only one signal peptide cleavage site was predicted in gp41. The online accessible tools MEME/MAST and ARNOLD could detect 30 putative host-specific promoters and 13 Rho-factor-independent terminators in the genome of Acibel004 (Table S3A and S3B). For eight genes, a conserved upstream sequence was found (Table S3C). The sequence was positioned between a promoter and the Shine-Dalgarno site of the following ORF and showed a consensus sequence of 5′-AAGCAAACAACTTAAAAGG-3′, which often included the putative ribosome binding site and is found 7 to 9 nucleotides upstream of the start codon. This conserved feature is limited to ORFs predicted in the middle region of the phage genome, and therefore may indicate the presence of a phage specific transcriptional regulator. The genome of Acibel004 is organized in several functional modules, including nucleic acids metabolism, early genes, tRNAs, DNA packaging, structural proteins and lysis cassette encoding modules (Fig. 5).

**Figure 6:**
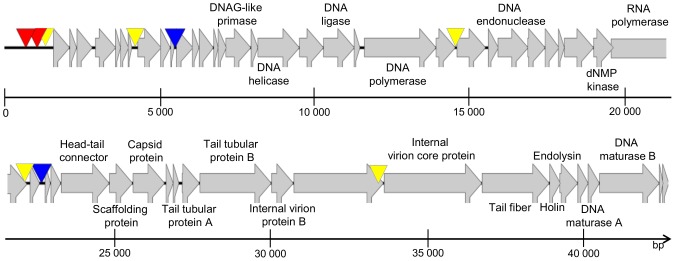
Genome map of Acibel007. Putative host-specific and phage-specific promoters and Rho-factor independent terminators are indicated with red, yellow and blue triangles, respectively.

The genome of Acibel007 contains 53 predicted ORFs (Fig. 6 and Table S4), all of them situated on the positive strand. No potential tRNA coding genes were found in the genome of Acibel007. Gene products of five ORFs may contain transmembrane domains but no signal peptide cleavage site was predicted in any of identified hypothetical proteins. Most of the identified proteins of Acibel007 showed similarity to proteins of phages belonging to the genus of the phiKMV-like phages of *Autographivirinae* subfamily. Bacteriophages belonging to the *Phikmvlikevirus* genus, are known to have direct terminal repeats (DTRs) which serve in the circularization of the phage genome after infection. Efforts to determine the direct terminal repeats in Acibel007 via direct sequencing were made, but failed.

Gene clustering and genome organization of Acibel007 are also similar to those typical for phages from the *Phikmvlikevirus* genus. In particular three distinct gene clusters could be identified: early genes, genes associated with DNA metabolism and genes coding for virion assembly, structural proteins and proteins involved in host lysis. The gene clusters are divided by 2 putative host-specific and 5 phage-specific promoters and 2 putative Rho-factor independent terminators (Fig. 6, Tables S5A, B and C). The early genes cluster in Acibel007 encodes 15 small proteins with no similarity to other gene products in Genebank database with only one exception for gp1. Gene product 1 corresponding proteins can be found in all phiKMV-like phages suggesting strongly conserved function of this protein in phage infection or host conversion.

Presence and order of the genes in the nucleic acids metabolism associated genes cluster correspond to others found in phiKMV like phages. However one major difference can be detected: gp31encoded in front of RNA polymerase gene has predicted function of dNMP kinase, an enzyme necessary for the rapid synthesis of the bacteriophage DNA in large amounts. This enzyme is not presented in any of phiKMV-like phages active against *P. aeruginosa* and other bacterial species except *A. baumannii* phages (phiAB1, AB3, Abp1). However the presence of dNMP is not unusual for the representatives of *T7likevirus* genus of *Autographivirinae* subfamily, the gene coding this putative enzyme can be found in the genomes of gh-1 and other similar to gh-1 phages. Gene product 31 of Acibel007 showed high similarity to the putative kinases encoded by the above *A. baumannii* phages and moderate similarity to kinase of T5 *Enterobacteria* phage, similarity is high especially close to enzyme’s N-termini region. Phage encoded dNMP kinases are mostly characterized by broad substrate specificity. Using either ATP or dATP as the phosphate donor, kinases of well-known T4 and T5 *Enterobacteria* phages catalyze the phosphorylation of 3 and 4 deoxynucleoside monophosphates, respectively [67]. Further tests need to be done to define whether Acibel007 encoded dNMP kinase also presents enzyme with broad substrate specificity.

One more difference in genome organization of phiKMV-like phages active against *A.baumannii* and other bacterial species, is presented by relocation of maturase genes from after lysis genes cassette position into the front of it. Also no Rz/Rz1 genes common part of lysis cassette in phiKMV-like phages [68] have been identified in Acibel007 and other related *Acinetobacter* phages yet.

### Proteome analysis of phages

SDS-PAGE and mass spectrometric analysis of purified phage particles of Acibel004 and Acibel007 were performed for the identification of structural phage proteins and to confirm gene predictions based on BLASTP homology search. After protein electrophoresis of the isolated Acibel004-proteins, ten protein bands could be visualized on gel with molecular weights ranging from 15 to 95 kDa. ESI-MS/MS analysis revealed the presence of 26 proteins (Table S6A). For 12 of these proteins, a structural function could be predicted based on homologous proteins found via BLASTP, while no function could be predicted for 11 other proteins. Seven proteins out of the above 11 located in the cluster of structural proteins will be assigned to be part of the phage particle structure based on these results. Three additional proteins out of total 26 (gp056, gp062 and gp066) are significantly present, but their predicted function is related to enzymatic activity of phages. The presence of these proteins in the structural proteome of phage Acibel004 could point out that they are needed in the early infection cycle and enter the cell together with the phage DNA upon infection of the bacterium. Only one protein (gp118) with a predicted structural function could not be identified by proteomics.

For Acibel007, 11 proteins were identified via ESI-MS/MS (Table S6B). For all except three of these proteins, a structural function was predicted via BLASTP homology search. These three unknown proteins (gp03, gp04, gp24) could be assigned to the early and nucleic acids metabolism regions of the phage genome. This can indicate that these proteins are essential in the first steps of the infection process, and are therefore included in the phage particle. One protein with a predicted structural function, (gp42) could not be detected via mass spectrometry.

### Comparative genomics and proteomics

The detailed genome analysis of Acibel004 showed that it represents a novel phage of *A. baumannii*. However, some of the structural proteins showed high similarity to the partially sequenced *A. baumannii* phage Abp53 [69]. Most of the annotated proteins encoded by Acibel004 also showed similarity to putative proteins of *P. aeruginosa* phages PAK-P1, PAK-P3 and KPP10, mostly including structural and hypothetical proteins with no defined functions Some enzymes such as DNA/RNA helicase, exodeoxyribonuclease and others also share similarity (Table S2). All three *P. aeruginosa* phages are strongly lytic phages and have been used in a number of phage therapy studies. In particular, phages PAK_P1 and PAK_P3 have been applied to combat lung infections in mice with successful therapeutic outcomes [70], [71], while KPP10 was also successfully used for treatment of gut-derived sepsis in mice [72]. The genome comparison performed by EMBOSS stretcher [50], [51] showed 47.1, 46.0 and 45.9% DNA homology of Acibel004 towards PAK-P1, PAK-P3 and KPP10, respectively. The genome organization of Acibel004 and the above phages carry some differences as well. Nucleic acids metabolism genes in Acibel004 are localized in a single large module while in the above phages it is divided by the region of early genes into two modules: a nucleotide metabolism module and a DNA replication/transcription associated genes modules [73]. In Acibel004 DNA metabolism associated and DNA packaging/structural proteins modules are separated by large region of more than 20 kbp length encoding proteins with no defined function, presumably the early genes region and tRNAs encoding genes. Most of the transcription regulatory elements are also accumulated in this region (Fig. 5).

**Figure 7:**
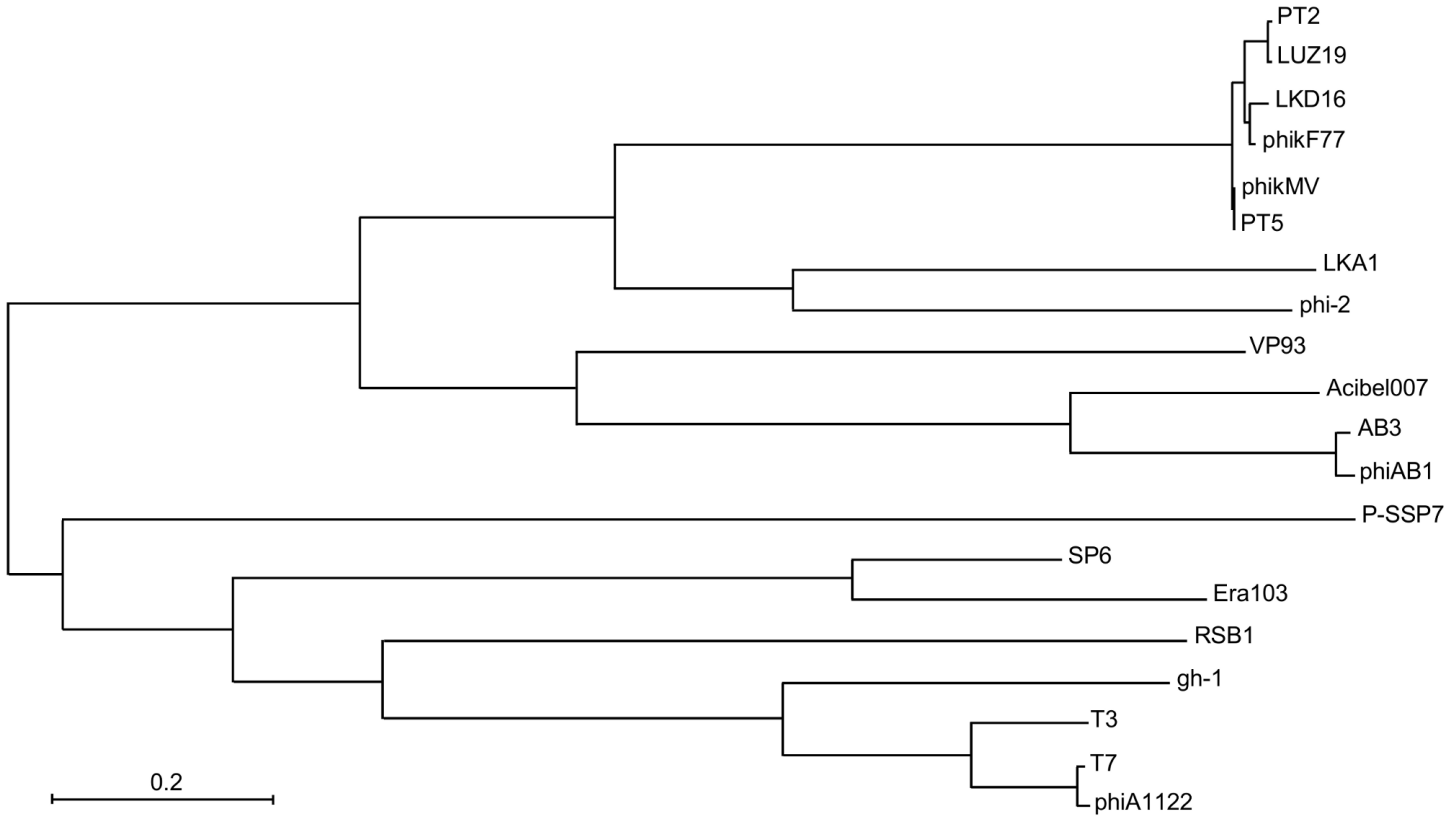
Phylogenetic relationship between RNA polymerases sequences of Acibel007 and 19 representatives of *Autographivirinae* subfamily.

Acibel007, like all other phages from the subfamily of the *Autographivirinae* encodes an RNA polymerase in its genome. For the phiKMV-likes, this RNA polymerase is situated at the end of the DNA replication region. Based on the sequence of RNA polymerases from the other 19 representatives of this subfamily, a phylogenetic tree was constructed (Fig. 7). As expected, Acibel007 grouped with the other *A. baumannii* phages included, i.e. phiAB1 and AB3, also showing strong relatedness to unclassified phiKMV-like phage VP93 active against *Vibrio parahaemolyticus*. At the DNA level, Acibel007 showed 54.7% similarity to phiAB1, and 46.2% to phiKMV.

No genes associated with pathogenicity/virulence or lysogeny (e.g. integrase encoding) were identified in the genomes of Acibel004 and Acibel007. The results mentioned in this study are clear indicators that both of these phages are good candidates for use in phage therapy against drug-resistant *A. baumannii* infections.

## Conclusions

The newly isolated *A. baumannii* phages Acibel004 and Acibel007 are characterized by a broad host range, a relatively low frequency of occurrence of phage-resistant mutant bacterial cells and infection parameters required by therapeutic phages in general. Close genetic relatedness of the studied phages to well-known groups of lytic phages together with the absence of the genes associated with lysogeny indicates a true virulent nature of these phages.

In conclusion, Acibel004 and Acibel007, based on thorough characteristic of their physiology, genome and proteome, can be regarded as phages with a high potential to be applied for bacteriophage-based therapy against infections caused by *A. baumannii* strains. Further *in vivo* safety and efficacy of these phages produced as a therapeutic preparation need to be evaluated in clinical trials.

## Supporting Information

Figure S1 **Dendrogram of fRFLP fingerprints of the 26** ***A. baumannii*** **phages.** Distance matrix calculated with 1 bp tolerance and 5% noise reduction using the dbp algorithm. Tree construction with Neighbor Joining.
(TIF)

Figure S2 **Activity of 26 newly isolated phages against the 34 strains of** ***Acinetobacter*** **spp.** Activity is defined by parallel streaks method expressing adsorption ability of phages.
(TIF)

Figure S3 **Codon frequency in** ***A. baumannii*** **ATCC 17978 and Acibel004.** Red markers indicate frequency of the codons matching anticodons of phage tRNAs in bacteria and phage.
(TIFF)

Table S1 **Characterization of 34 strains of** ***Acinetobacter*** **spp. used in the study.**
(XLSX)

Table S2 **Annotation table of Acibel004 genome.**
(XLSX)

Table S3 **Transcription regulators of Acibel004 genome.** A) Putative host-specific σ^70^-dependent promoters of Acibel004; B) Putative Rho-factor-independent terminators of Acibel004; C) Putative conserved regulators of Acibel004.
(DOCX)

Table S4 **Annotation table of Acibel007 genome.**
(XLSX)

Table S5 **Transcription regulators of Acibel007.** A) Putative host-specific σ^70^-dependent promoters of Acibel007; B) Putative phage-specific promoters; C) Putative Rho-factor-independent terminators of Acibel007.
(DOCX)

Table S6 **Characterization of phage structural proteins identified by ESI-MS/MS.** A) Structural proteins of Acibel004; B) Structural proteins of Acibel007.
(DOCX)
