# Supplementary figures and images for: Characterization of Newly Isolated Lytic Bacteriophages Active against Acinetobacter baumannii
Source: PLoS One. 2014 Aug 11;9(8):e104853. doi: 10.1371/journal.pone.0104853 (PMC4128745; doi:10.1371/journal.pone.0104853)

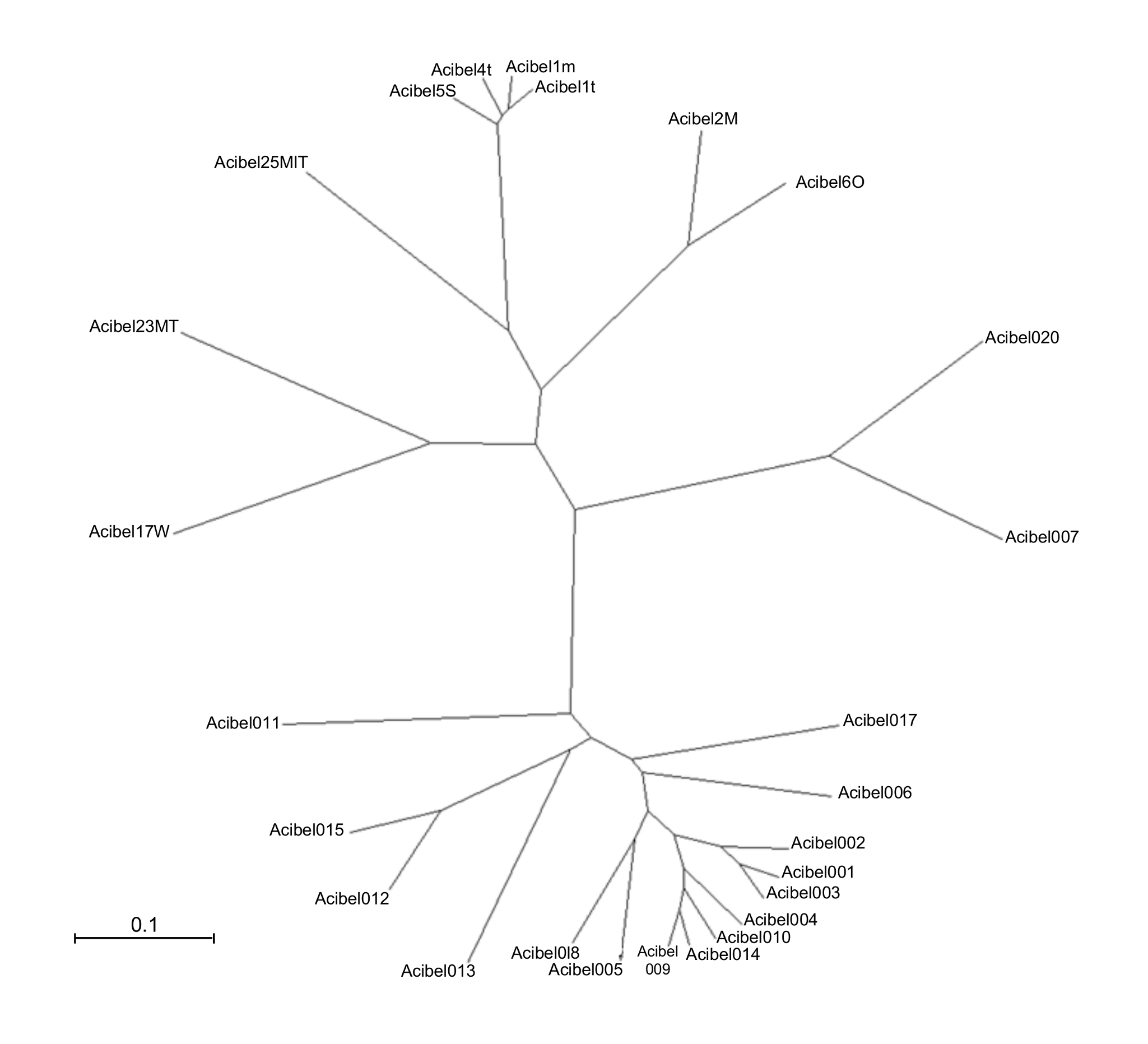

Supplement: Figure S1 — Dendrogram of fRFLP fingerprints of the 26 A. baumannii phages. Distance matrix calculated with 1 bp tolerance and 5% noise reduction using the dbp algorithm. Tree construction with Neighbor Joining. (TIF) [file pone.0104853.s001.tif]

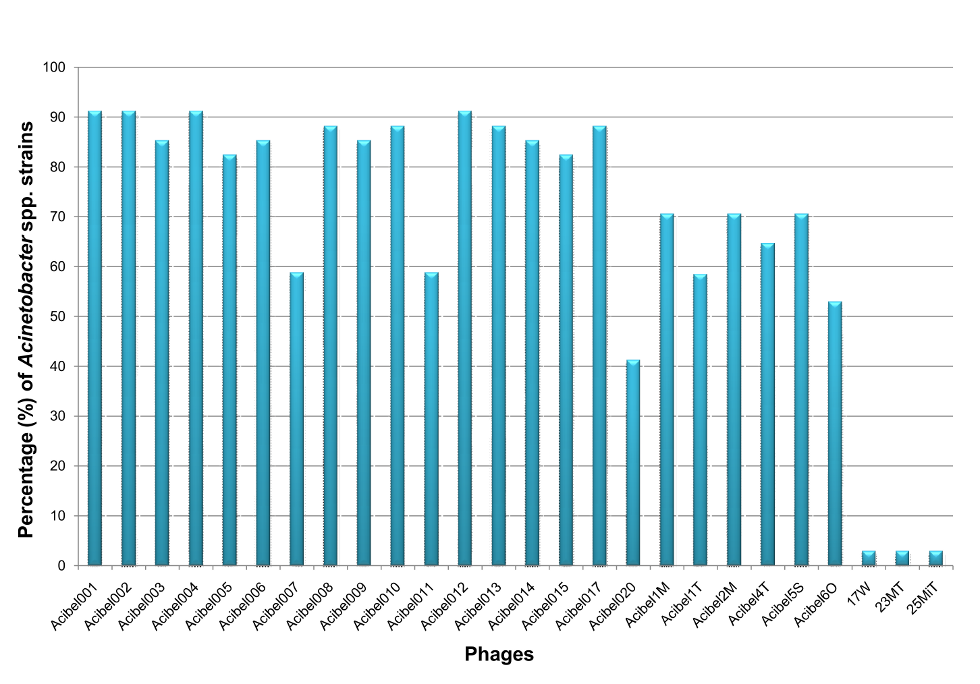

Supplement: Figure S2 — Activity of 26 newly isolated phages against the 34 strains of Acinetobacter spp. Activity is defined by parallel streaks method expressing adsorption ability of phages. (TIF) [file pone.0104853.s002.tif]

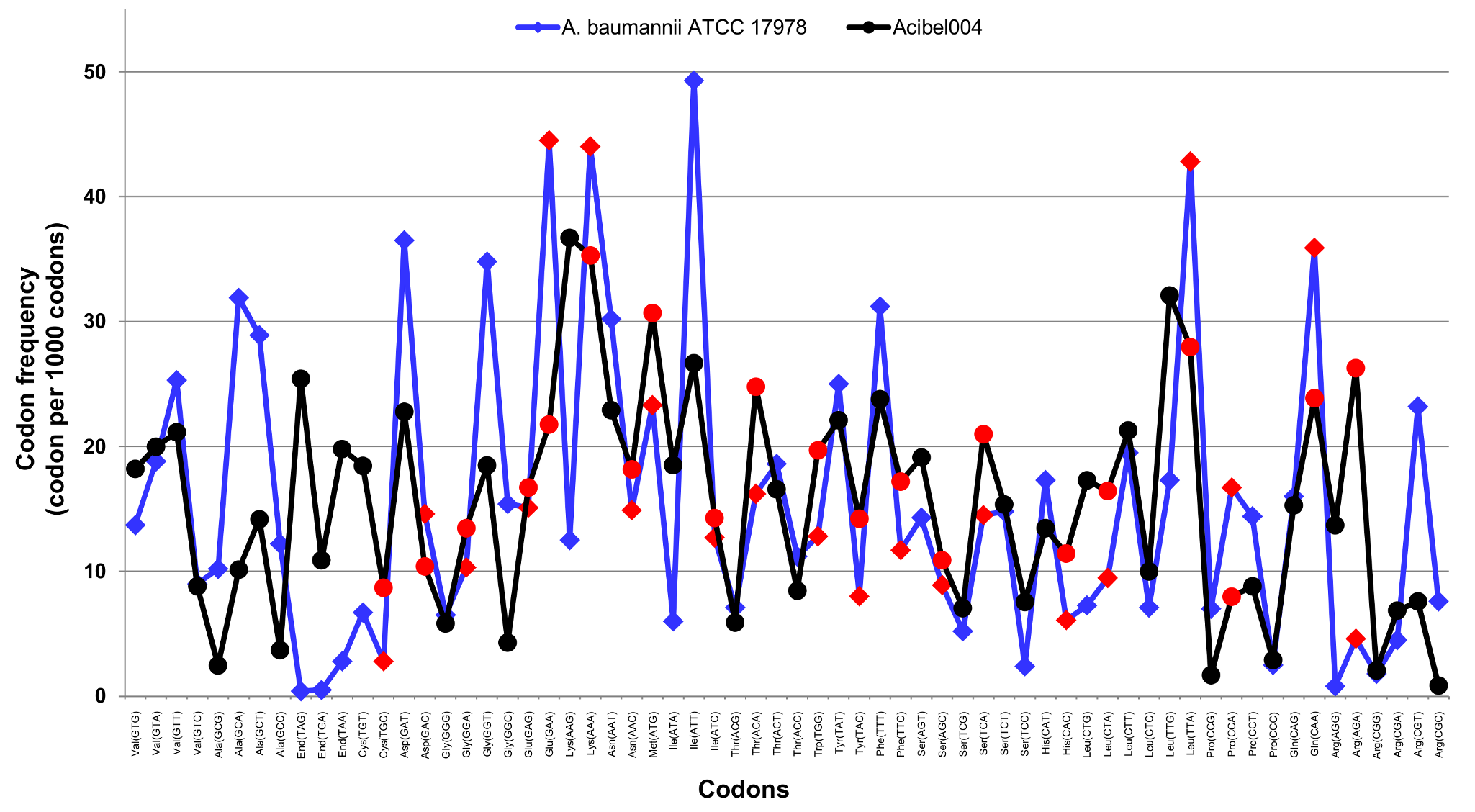

Supplement: Figure S3 — Codon frequency in A. baumannii ATCC 17978 and Acibel004. Red markers indicate frequency of the codons matching anticodons of phage tRNAs in bacteria and phage. (TIFF) [file pone.0104853.s003.tiff]
