# Supplementary material for: Characterization of Newly Isolated Lytic Bacteriophages Active against Acinetobacter baumannii
Source: PLoS One. 2014 Aug 11;9(8):e104853. doi: 10.1371/journal.pone.0104853 (PMC4128745; doi:10.1371/journal.pone.0104853)
Supplement: Table S3 — Transcription regulators of Acibel004 genome. A) Putative host-specific σ70-dependent promoters of Acibel004; B) Putative Rho-factor-independent terminators of Acibel004; C) Putative conserved regulators of Acibel004. (DOCX) [file pone.0104853.s006.docx]

**Table S3A.** Putative host-specific σ^70^-dependent promoters of Acibel004

| **N** | **Position** | **Strand** | **Sequence** |
| --- | --- | --- | --- |
| P01 | 2546_2650 | + | TAACAGTTGATTAACGTAGCTGGTGTGTGTATAATACATGCACTG |
| P02 | 23057_23103 | + | TAAAGTTGTTGACCTGTTATATGCTTGTGTATATAATGGGTCTATTC |
| P03 | 27845_27890 | + | AATATTTGTTGACAACCAATTAATTTAATATATAATTAAATCCATA |
| P04 | 33278_33324 | + | AATAGTTGTTGACAACATGCCTTGAGGGTGGCATAATACCCTCATCA |
| P05 | 40683_40727 | + | TAAGGGTTGACATACAGCCCTTGTCTCTATAAAATGAACCACATA |
| P06 | 41460_41503 | + | AGGTGGTTGCCTTTTTTATTGCCTATGTTATAATAAATTAAATT |
| P07 | 43345_43390 | + | GTGTGGTTGACATAGGTTAGCCATCCACTATATAATAGAACTCTTA |
| P08 | 43987_44030 | + | AAGGTATTGCAAAAATAATTTATTCCTATAGAATGGCATCCATA |
| P09 | 45142_45186 | + | AAAGAGTTGACACAATAGAAAAACACGCCCATAATGCACCACATA |
| P10 | 45531_45575 | + | AAACGCTTGACACATTAACTTAATATCTGTACTATGAACCACATA |
| P11 | 45979_46023 | + | AAACACTTGACTAGGGTATCAAAAAGCTTTAATATTCTAGTCATA |
| P12 | 47426_47471 | + | TCTTCATTGCCGTTCTAAGACACGTTTACCTATAAGGGCTACAACC |
| P13 | 47608_47652 | + | AAGGGCTTGACAGTATTGTACAAGTCCTTTAATATGGCACTCATA |
| P14 | 47770_47817 | + | TTATGATTGGTTAATATAATGGGAAAGGTTGATATAATCAGAGCAAAG |
| P15 | 48356_48401 | + | AAAAGCTTGCTTTTAGCATGAATTTAAAGTTATAATGAGTTCATGC |
| P16 | 48882_48927 | + | AAGGTATTGCAGAATGATTAAATTTCTTGCTATACTTTGCCTGTCG |
| P17 | 49037_49081 | + | ATTTACTTGACACTATAGATTTATTTCTTTAGAATGTAACACATA |
| P18 | 49848_49894 | + | ATTCACTTGACAAGGAAGTCAAACAAAGCCATAATAGGCACATACCA |
| P19 | 50986_51029 | + | TAGGGCTTGCAATTACTTTAAACGTCTATATAATGAAAGACACA |
| P20 | 51721_51766 | + | CAAGTGTTGCCGTTATCTCATTTCATGAGAATTAATATTTAAATAA |
| P21 | 52244_52287 | + | TAGAGTTTGTCAAGCACTTTATTCAGTTAAATTTCAGGATCACA |
| P22 | 52361_52405 | + | AATGTATTGCATTGAGAGGATAATTCAAGTATAATACTTTAGACT |
| P23 | 52465_52508 | + | GTCGATTTGTCAAGTCTTTTTTATATGAATTATTCTTATAAGGA |
| P24 | 52588_52632 | + | AATAAATTGACACGGCGTATGGAAATAGGCATAATAAGCACATAC |
| P25 | 59276_59233 | - | TAAGTCTTGACTTCTACATGATGTCATGTATAATACAACCTCAT |
| P26 | 59313_59359 | + | ATAAACTTGACAAATCAATATTTCATGGTACTATAACTTTAGTTATC |
| P27 | 59561_59609 | + | ATTGTCTTGACTTTTCAGGAATCTGTGGTATAGTATTATCATGAATTAG |
| P28 | 99307-99263 | - | AAGGGTTTGATTTTTAAGAACGAAAGCATAGAATAGCGTCATCA |
| P29 | 99519_99566 | + | CAAATGTGTTGACTTAGAATATTATTATTGTATATTAGATATATAAAT |
| P30 | 99635_99678 | + | AAAGTTTTGTAACCAGTTACACGACTTATACAATTGTGTCTATA |

**Table S3B.** Rho-factor-independent terminators of Acibel004

| **N** | **Position** | **Strand** | **Sequence** | **ΔG (kcal/mol)** |
| --- | --- | --- | --- | --- |
| T01 | 12272-12300 | + | TCCAGCTTCGGCTGGATTTTTTAGTTTAT | -12.90 |
| T02 | 15296-12333 | + | GGGACATATCCGAAAGGGTGTGTCCCTTTTCTGTTTTT | -11.20 |
| T03 | 17845-17881 | + | GGCAATTATCTTTAATTAGGTAGTTGCCTTTTTTATT | -7.43 |
| T04 | 27810-27838 | + | GGGAGGGCTTCGGCTCTCCCTTTTTTATT | -18.7 |
| T05 | 40686-40712 | + | GGGTTGACATACAGCCCTTGTCTCTAT | -8.70 |
| T06 | 41447-41479 | + | GGCAATCATCATAAGGTGGTTGCCTTTTTTATT | -11.10 |
| T07 | 44968-44995 | + | GAGGCGCAAGCCTCTTTTTTGTTGTCTT | -12.80 |
| T08 | 57335-57298 | - | GACCTGAAGCGTTAGACTGCTTGCAGGTCTTTTTTGTT | -8.43 |
| T09 | 64218-64241 | + | GCCCATTAGTGGGCTTTATTTTTT | -8.50 |
| T10 | 70745-70776 | + | GGGGACATCGTTTGATGTCCTCTTTTTCCCTT | -11.90 |
| T11 | 96888-96862 | - | GGGAGCTTATGCTCCCTTTTCTTTTAT | -12.80 |
| T12 | 96873-96896 | + | GGGAGCATAAGCTCCCTATTTTTT | -13.00 |
| T13 | 97405-97378 | - | GGGGAGCTTCGGCTCTCCTTTTTATTGT | -15.20 |

**Table S3C.** Putative conserved regulators of Acibel004

| **Regulator** | **Start** | **End** | **Strand** | **Sequence** |
| --- | --- | --- | --- | --- |
| R01 | 44033 | 44051 | + | AAGCAAACAACTTAAAAGG |
| R02 | 45578 | 45596 | + | CAGCAAACAACTTAAAAGG |
| R03 | 46026 | 46042 | + | AAGCAAACAACGAAAGG |
| R04 | 47655 | 47673 | + | AAGCAAACAACTTAAAAGG |
| R05 | 48410 | 48428 | + | AAACAAACAACTTAAAAGG |
| R06 | 49084 | 49102 | + | AAGCAAACAACATACAAGG |
| R07 | 50630 | 50648 | + | AATCAAACAACTTAAAAGG |
| R08 | 52634 | 52654 | + | AAGCAAACAACATATTAAAGG |
