# Supplementary material for: Characterization of Newly Isolated Lytic Bacteriophages Active against Acinetobacter baumannii
Source: PLoS One. 2014 Aug 11;9(8):e104853. doi: 10.1371/journal.pone.0104853 (PMC4128745; doi:10.1371/journal.pone.0104853)
Supplement: Table S5 — Transcription regulators of Acibel007. A) Putative host-specific σ70-dependent promoters of Acibel007; B) Putative phage-specific promoters; C) Putative Rho-factor-independent terminators of Acibel007. (DOCX) [file pone.0104853.s008.docx]

**Table S5A.** Putative host-specific σ^70^-dependent promoters of Acibel007

| **N** | **Position** | **Strand** | **Sequence** |
| --- | --- | --- | --- |
| P01 | 689-734 | + | ATAGAGTGTTGACATAGTTTTTAAACTGTATATTATGACAACCATA |
| P02 | 1052-1098 | + | ATAAAGTGTTGACAACAAAATCAGATATGATTAAGATGCCAACCATA |

**Table S5B.** Putative phage-specific promoters of Acibel007

| **N** | **Position** | **Strand** | **Sequence** |
| --- | --- | --- | --- |
| P01 | 1316-1351 | + | CTGTGAGGCTGTACTCACAGTTAATTCCAATACATA |
| P02 | 4240-4275 | + | TCGTGAGTCTGTACTCACAATTATATTTCGCATTTA |
| P03 | 14517-14552 | + | AGTTTAACCTGTACTCACAACTCAATTCTTAAATTT |
| P04 | 22008-22043 | + | CTTGGACTCTGTACTCACAGCTCAATTTATGTTTTG |
| P05 | 28790-28825 | + | TAACTCGACTGTACTCACACCTAAATCTGCGGTGAT |

**Table S5C.** Putative Rho-factor-independent terminators of Acibel007

| **N** | **Position** | **Strand** | **Sequence** | **ΔG (kcal/mol)** |
| --- | --- | --- | --- | --- |
| T01 | 5487-5519 | + | CAGCACTTCGATGAGGTGCTGTTAGTATTTAAT | -11.00 |
| T02 | 22374-22415 | + | GCCCTTGAGCCTAACCGCTTGAGGGCTTTTTTTTTT | -10.50 |
