# Supplementary material for: Characterization of Newly Isolated Lytic Bacteriophages Active against Acinetobacter baumannii
Source: PLoS One. 2014 Aug 11;9(8):e104853. doi: 10.1371/journal.pone.0104853 (PMC4128745; doi:10.1371/journal.pone.0104853)
Supplement: Table S6 — Characterization of phage structural proteins identified by ESI-MS/MS. A) Structural proteins of Acibel004; B) Structural proteins of Acibel007. (DOCX) [file pone.0104853.s009.docx]

**Table S6A.** Structural proteins of Acibel004 identified by ESI-MS/MS

| **Protein name** | **Predicted function** | **Protein molecular weight (Da)** | **Protein identification probability** | **Number of unique peptides** | **Percentage sequence coverage** | **Best Peptide identification probability** |
| --- | --- | --- | --- | --- | --- | --- |
| ORF007 | Unknown | 42 612.80 | 100.00% | 7 | 20.99% | 95.00% |
| ORF015 | Unknown | 24 264.20 | 100.00% | 3 | 18.90% | 95.00% |
| ORF020 | Unknown | 20 947.60 | 100.00% | 10 | 54.60% | 95.00% |
| ORF043 | Unknown | 19 597.20 | 100.00% | 3 | 20.80% | 95.00% |
| ORF056 | RnlB RNA ligase 2 | 42 801.10 | 100.00% | 3 | 8.44% | 95.00% |
| ORF062 | Putative Nicotinate Phosphoribosyltransferase | 70 695.20 | 100.00% | 3 | 6.00% | 95.00% |
| ORF066 | PspA/IM30 Family Protein | 25 226.00 | 99.80% | 2 | 11.90% | 95.00% |
| ORF106 | Unknown | 15 670.90 | 100.00% | 3 | 26.84% | 95.00% |
| ORF119 | Unknown | 55 382.60 | 100.00% | 13 | 27.21% | 95.00% |
| ORF121 | Unknown | 18 166.50 | 100.00% | 11 | 64.40% | 95.00% |
| ORF122 | Major Capsid Protein | 40 885.80 | 100.00% | 27 | 77.50% | 95.00% |
| ORF123 | Tail Component | 236 725.01 | 100.00% | 3 | 2.32% | 95.00% |
| ORF129 | Unknown | 25 502.70 | 100.00% | 4 | 21.88% | 95.00% |
| ORF130 | Putative Structural Protein | 39 051.10 | 100.00% | 18 | 70.30% | 95.00% |
| ORF131 | Putative Structural Protein | 19 264.90 | 100.00% | 6 | 44.10% | 95.00% |
| ORF132 | Putative Structural Protein | 20 542.00 | 100.00% | 2 | 11.64% | 95.00% |
| ORF134 | Putative Structural Protein | 17 836.90 | 100.00% | 4 | 27.70% | 95.00% |
| ORF136 | Phage Tape Measure Protein | 72 049.50 | 100.00% | 6 | 13.40% | 95.00% |
| ORF137 | Unknown | 32 003.80 | 100.00% | 3 | 16.50% | 95.00% |
| ORF140 | Putative Baseplate Protein | 28 118.00 | 100.00% | 2 | 13.72% | 95.00% |
| ORF141 | Unknown | 14 131.90 | 100.00% | 2 | 19.20% | 95.00% |
| ORF142 | Putative Baseplate Component | 52 264.80 | 100.00% | 8 | 28.80% | 95.00% |
| ORF143 | Tail component | 38 005.30 | 100.00% | 4 | 16.07% | 95.00% |
| ORF145 | Putative Tail Fiber Protein | 39 951.20 | 100.00% | 6 | 30.10% | 95.00% |
| ORF148 | Putative Tail Fiber Protein | 42 470.30 | 100.00% | 12 | 44.50% | 95.00% |
| ORF150 | Putative Lysozyme | 22 755.40 | 99.90% | 2 | 13.60% | 95.00% |

**Table S6B.** Structural proteins of Acibel007 identified by ESI-MS/MS

| **Protein name** | **Predicted function** | **Protein molecular weight (Da)** | **Protein identification probability** | **Number of unique peptides** | **Percentage sequence coverage** | **Best Peptide identification probability** |
| --- | --- | --- | --- | --- | --- | --- |
| ORF03 | Unknown | 18 646.80 | 99.50% | 2 | 10.80% | 95.00% |
| ORF04 | Unknown | 24 222.20 | 99.80% | 2 | 11.50% | 95.00% |
| ORF24 | Hypothetical Protein | 33 470.60 | 100.00% | 3 | 10.40% | 95.00% |
| ORF35 | Putative Structural Protein | 11 571.30 | 100.00% | 6 | 41.10% | 95.00% |
| ORF36 | Putative Head-Tail Connector Protein | 58 830.90 | 100.00% | 2 | 5.04% | 95.00% |
| ORF38 | Putative Capsid Protein | 37 406.70 | 100.00% | 23 | 76.30% | 95.00% |
| ORF41 | Putative Tail Tubular Protein A | 21 380.00 | 100.00% | 7 | 51.40% | 95.00% |
| ORF43 | Internal virion protein B | 24 178.00 | 100.00% | 2 | 20.51% | 95.00% |
| ORF44 | Putative Structural Protein | 106 012.50 | 100.00% | 3 | 5.39% | 95.00% |
| ORF45 | Putative Internal Virion Core Protein | 114 947.10 | 100.00% | 8 | 11.50% | 95.00% |
| ORF46 | Putative Tail Fiber | 78 676.40 | 99.80% | 2 | 6.63% | 95.00% |
